# *Anaplasma* and *Theileria* Pathogens in Cattle of Lambwe Valley, Kenya: A Case for Pro-Active Surveillance in the Wildlife–Livestock Interface

**DOI:** 10.3390/microorganisms8111830

**Published:** 2020-11-20

**Authors:** Michael N. Okal, Brenda Kisia Odhiambo, Peter Otieno, Joel L. Bargul, Daniel Masiga, Jandouwe Villinger, Shewit Kalayou

**Affiliations:** 1 International Centre of Insect Physiology and Ecology (icipe), P.O. Box 30772-00100 Nairobi, Kenya; mnyanganga@icipe.org (M.N.O.); bodhiambo@icipe.org (B.K.O.); podhiambo@icipe.org (P.O.); jbargul@icipe.org (J.L.B.); dmasiga@icipe.org (D.M.); 2 Department of Biochemistry, Jomo Kenyatta University of Agriculture and Technology, P.O. Box 62000-00200 Nairobi, Kenya

**Keywords:** tick-borne pathogens, *Anaplasma*, *Theileria*, zebu cattle, wildlife–livestock interface, Kenya

## Abstract

Tick-borne pathogens (TBPs) are major constraints to livestock production and a threat to public health in Africa. This cross-sectional study investigated the risk of infection with TBPs in cattle of Lambwe Valley, Kenya. Blood samples of 680 zebu cattle from 95 herds in six geospatial clusters within 5 km of Ruma National Park were screened for bacterial and protozoan TBPs by high-resolution melting analysis and sequencing of PCR products. We detected *Anaplasma bovis* (17.4%), *Anaplasma platys* (16.9%)*, Anaplasma marginale* (0.6%), *Theileria velifera* (40%), and *Theileria mutans* (25.7%), as well as an *Anaplasma* sp. (11.6%) that matched recently reported *Anaplasma* sp. sequences from Ethiopia. *Babesia*, *Rickettsia,* and *Ehrlichia* spp. were not detected. The animal and herd-level prevalences for TBPs were 78.5% (95% confidence intervals (CI): 75.3, 81.5) and 95.8% (95% CI: 91.8, 99.8), respectively. About 31.6% of cattle were co-infected with 13 combinations of TBPs. The prevalence of TBPs differed between clusters and age, but the risk of infection was not associated with sex, herd size, or the distance of homesteads from Ruma. This study adds insight into the epidemiology of TBPs around Ruma and highlights the need for proactive surveillance of TBPs in livestock–wildlife interfaces.

## 1. Introduction

Wildlife–livestock interfaces, or areas where game and domesticated animals co-habit [1], support a plethora of interactions that can be of epidemiological concern [2]. Such interfaces are a haven for ticks and many other arthropod vectors of diseases maintained by wildlife [1]. Global trends suggest that emerging infectious diseases are on the rise, with up to 80% of animal pathogens and 71.8% of emerging zoonoses in some regions predicted to have a wildlife component [3,4]. Over the past two decades, there has been a heightened incidence of ticks and tick-borne pathogens (TBPs) across a wide geographical spread [3] and new tick-borne disease agents often discovered [5]. Many of these infectious diseases transmitted by ticks between domestic and wild animals represent emerging and re-emerging burdens to global public health, economies, and the conservation of biodiversity [2,6]. Astonishingly, there is limited information on the diversity and prevalence of TBPs in many areas, including the wildlife–livestock interfaces. Where available, data are dated and might not be beneficial for the strategic management and control of TBPs [6].

In sub-Saharan Africa, theileriosis, ehrlichiosis, babesiosis, and anaplasmosis spread by ticks are the leading causes of losses in livestock production [7]. Several TBPs have been identified in wildlife [8,9], ticks [9,10,11], and livestock, including small ruminants kept by nomads [12]. Coexistence with wildlife can increase the risk of TBPs in livestock and man [13]. The most important tick-borne disease in sub-Saharan Africa, the corridor disease caused by *Theileria parva*, is believed to have co-evolved with the African cape buffalo, a host immune to the pathogen, before “jumping hosts” to cattle [14]. More recently, studies have reported diverse TBPs in Kenya, including a novel *Rickettsia* sp. identified in ticks in the coastal Shimba Hills National Reserve [5]. A prevalence of up to 5.5% spotted fever group rickettsioses was found in wildlife in Laikipia and the Maasai Mara National Reserve [8,9]. Tortoises in Baringo County and monitor lizards in Homa Bay County have been implicated as reservoirs of *Ehrlichia ruminantium* and *Ehrlichia canis*, respectively [10]. Similarly, Bunyamwera and West Nile viruses were identified from ticks collected from livestock and wildlife in Ijara District, northern Kenya [15]. These findings demonstrate the importance of wildlife in the circulation of diseases.

In Kenya, national parks, reserves, and conservancies account for about 11% of the country’s landmass [16]. This is the direct result of wildlife conservation initiatives during the 1970s that led to the conversion of large portions of arid and semi-arid ecosystems, then considered to have little agricultural potential, into protected areas [1]. Ruma National Park, which occupies a third of Lambwe Valley in western Kenya, is a wildlife protected area where significant changes in land use have occurred over the last two decades [17]. In the last half century, intensive multiagency tsetse fly control activities in Lambwe Valley resulted in a marked decline in the incidence of trypanosomiasis [18] and contributed to the local elimination of human African sleeping sickness. The effective interventions impacted land use so that cultivated land increased by 22% over the last three decades [17]. Human populations on the periphery of the protected area have increased significantly [18], leading to an equivalent increase in livestock [19]. Such trends often increase the interactions between livestock, humans, and wildlife and may create a complex disease environment that leads to spill-over of diseases from wildlife to livestock and humans, and vice versa [20]. Despite the Ruma wildlife–livestock interface being a unique epidemiological niche of concern, empirical data on the epidemiology of TBPs circulating in livestock in the valley is limited.

Recently, smallholder farmers who reside within Lambwe Valley complained of episodes of outbreaks in livestock, causing significant fatality. Such reports also identified the presence of ticks and clinical signs compatible with haemoparasite infection in cattle. We hypothesised that the cattle were infected with TBPs and that cattle populations at the wildlife–livestock interface were at increased risk of TBP infection. We conducted a molecular epidemiological study to investigate the prevalence of TBPs and identify putative risk factors associated with exposure in cattle reared under a traditional system in the Lambwe Valley ecosystem.

## 2. Materials and Methods

### 2.1. Study Area

**Figure 1:**
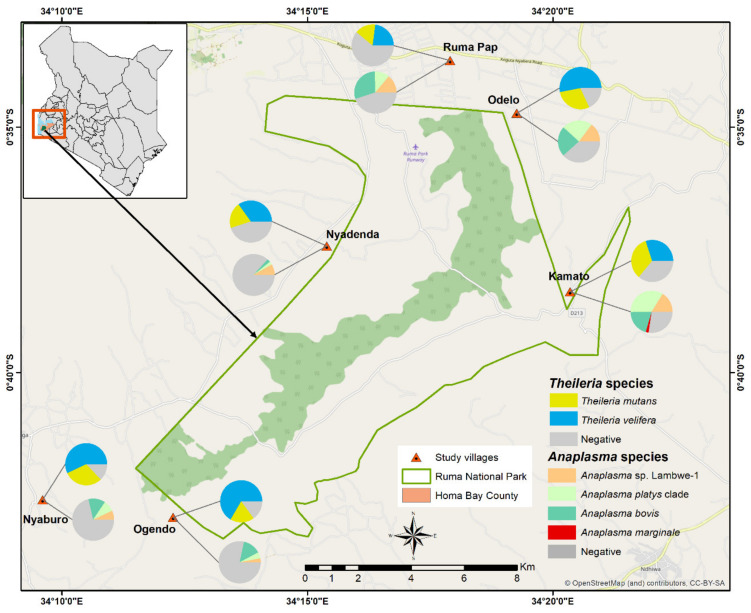
Livestock sampling and tick-borne pathogen (TBP) prevalence map across the the study villages of Lambwe Valley in western Kenya.

The study was carried out in Lambwe Valley, in village clusters around the Ruma National Park (Figure 1). Lambwe Valley (latitude 0°38′ 35.52″ S, longitude 34°16′48″ E) is 1200–1600 m above sea level and is infested by tsetse flies, the major vector for African trypanosomiasis. The terrain consists of rolling grasslands with open woodland and thickets dominated by acacia and a variety of grass species. The soil is predominantly black cotton, and the climate is hot and humid with an average annual air temperature of 25 °C. Rainfall is bimodal, peaking in April–June and September–November. The annual dry and hot period is from January to March [21]. The community in Lambwe Valley practice subsistence farming, fishing, and animal husbandry. In the park, the main grazing and browsing wild ruminant populations consist of roan antelope (*Hippotragus equinus langheldi*), Jackson’s hartebeest (*Alcelaphus buselaphus Jacksonii*), oribi (*Ourebia ourebi*), and Rothschild giraffes (*Giraffa camelopardalis rothschildi*). The indigenous zebu cattle breed is the dominant domestic species followed by small ruminants. Livestock graze in the open fields extending to the park’s fence, thus creating an interface for interaction between wildlife and livestock.

### 2.2. Study Design and Sample Size Determination

We used a cross-sectional design with stratified one-stage cluster sampling. All zebu cattle within a 5 km radius from the park’s fence were defined as the study population. Construction of a sampling frame was not possible due to lack of reliable cattle demographic data. Therefore, we defined clusters based on the coordinates of a map grid (3 km^2^), which later was named after the nearest village (Figure 1). Data were collected between December 2018 and February 2019. Indigenous zebu cattle that were at least one year old and managed in smallholder farms in the wildlife–livestock interface of the Lambwe Valley were considered eligible for the study. This age category is likely to interact with other herds or wildlife at watering points and/or during grazing and is thus amenable to TBP surveillance in the area. Sample sizes were determined based on one-stage cluster sampling using previously described methods [22,23,24].

The sample size to estimate the prevalence with a specified precision is given by:

(1) $$N\;=\;\mathrm{gc}\;=\;\frac{P\left(1-P\right)D}{{\mathrm{SE}}^{2}}$$

where N is the sample size, P is the prevalence, D is the design effect, SE is the standard error of an estimated proportion P, g is the average number of animal sampled per cluster, and c the number of clusters sampled [22]. The design effect was given by the formula,

(2) $$D\;=\;1\;+\;\left(g-1\right)\mathrm{ICC}.$$

We restricted the number of sampled villages (clusters) to 20% (c = 6) of the total 35 clusters, which were selected randomly, but accounting for accessibility. The intra-cluster correlation coefficient (ICC) is a measure of homogeneity of clustered data. We used the ICC value of 0.04 for *Anaplasma marginale* from previous studies [25]. Considering the possibility to collect a maximum of 100 samples by a team of six people per day per cluster, D was calculated as 5 (Equation (2)). Prevalence information for TBPs is limited in the study area. Therefore, the study assumed a prevalence (P) of 50% for the pathogens. With an expected prevalence of 50%, a cluster size of 6 and the desired precision of 5%, a total sample size of 500 animals was estimated. The study was announced to farmers for sensitisation through local radio, and we accounted for a 25% non-response rate. To consider the sample size for the non-response rate:

(3) $$N\;=\;\frac{\left(\mathrm{Sample}\;\mathrm{size}\;\mathrm{calculated}\right)}{\left(1-\mathrm{nonresponse}\;\mathrm{rate}\right)}$$

The above equations gave an estimated sample size of 667, and the final dataset consisted of a total of 680 cattle. 

Epidemiological data on potential risk factors were collected from each sampled household using a questionnaire that captured sex, herd size, herd husbandry practices, grazing system, commonly used acaricides, and the frequency of application. 

### 2.3. Ethical Approval

This study strictly adhered to the experimental guidelines and procedures approved by the Institutional Animal Care and Use Committee at International Centre of Insect Physiology and Ecology (*icipe)* and Kenya’s animal welfare laws under the Veterinary Surgeons and Veterinary Para-professionals Act, 2011 (Cap. 366). Blood samples were collected only after receiving informed verbal consent from cattle keepers. Verbal rather than written consent was adopted as the pragmatic approach because all farmers were neither able to read nor write. Blood samples were collected by an experienced veterinarian with the aim of minimising pain and discomfort.

### 2.4. Blood Sample Collection and Processing

Blood samples were collected from the marginal ear vein to assess the anaemia status of each animal by measuring packed cell volume (PCV) using the micro-haematocrit method. Briefly, capillary blood was drawn into ethylenediaminetetraacetic acid (EDTA) microhematocrit tubes and centrifuged at 13,000× *g* for 10 min. PCV levels were calculated using the Hawksley haematocrit reader. For molecular detection of pathogens, about 4 mL of blood was collected from the jugular vein of each cattle using sterile vacutainer needles and EDTA vacutainers. Each tube was identified by a unique animal’s ID number. Blood in the EDTA vacutainers were transferred into uniquely-labelled cryovials, stored in liquid nitrogen, and transported to *icipe*’s Martin Lüscher-Emerging Infectious Diseases (ML-EID) Laboratory for molecular analysis.

### 2.5. PCR and High-Resolution Melting (HRM) Analysis

DNA was extracted from blood samples using the Bioline Isolate II genomic DNA kit (Meridian Life Science, Memphis, TN, USA) as described by the manufacturer and screened for the presence of *Anaplasma*, *Theileria*, *Ehrlichia*, *Rickettsia,* and *Babesia* spp. by PCR using single-plex genus-specific primers, as shown in Table 1. The 10 µL PCR volumes consisted of 2 µL of the extracted DNA template, 2 µL 5× HOT FIREPol^®^ EvaGreen HRM Mix (Solis BioDyne, Tartu, Estonia), 0.5 µL each of the 10 µM forward and reverse primers for the respective genus-specific reactions, and 5 µL PCR water. Each assay on different pathogens (*Ehrlichia* spp., *Rickettsia* spp., *Anaplasma* spp., *Theileria* spp., and *Babesia* spp.) had a set of known positive controls and a negative control (nuclease-free water) All PCR-HRM reactions were done in the Rotor-Gene Q 5-Plex HRM capable thermocycler (Qiagen, Hannover, Germany). The amplifications involved touchdown PCR conditions including an initial activation of the polymerase enzyme at 95 °C for 15 min, followed by 35 cycles of denaturation at a temperature of 9 °C for 20 sec. The 25 sec per cycle annealing temperature was reduced by 1 °C each cycle during the first ten cycles from 65°C to 55 °C, and followed by extension at 72 °C for 25 s per cycle and a final extension step for 3 min at 72 °C. Then, the products were maintained for 3 min at 72 °C before proceeding with HRM analysis, in which the temperature was gradually increased from 75 °C to 95 °C with fluorescence acquisition after 2 s at 0.1°C increments. Changes in fluorescence with change in temperature (dF/dT) were recorded and plotted. Melt curves were visualised and analysed on Rotor-Gene Q software version 2.3.1 (Qiagen, Hannover, Germany). The graphs were normalised between 100 and 0% fluorescence. The positive PCR samples were identified by comparing the different melting profiles with the positive controls using the HRM analysis software version 2.1.0 (Qiagen, Hannover, Germany). The PCR products were run in 2% agarose gels and purified using ExoSap-IT (USB Corporation, Cleveland, OH, USA) as per the manufacturers’ instruction and then Sanger sequenced at Macrogen Inc. (Seoul, Korea). Gene sequences produced from this study were deposited in the GenBank database of the National Centre for Biotechnology Information.

### 2.6. Nested PCR for Anaplasma-Positive Samples

To amplify larger DNA fragments of *Anaplasma*, we performed nested PCR targeting family Anaplasmataceae 16S rRNA genes [27] in samples with *Anaplasma* amplicons that had unique HRM profiles. Amplification was done in a TM100 thermal cycler (BioRad, Hercules, CA, USA) in 10 µL PCR volumes comprised of 2 µL of 5X HOT FIREPol^®^ Blend Master Mix (Solis BioDyne, Tartu, Estonia), 0.5 µL each of the 10 µM forward and reverse primers, 2 µL template, and 5 µL PCR-grade water. Primary PCR amplifications using the EHR16SD and pH1522 primers (Table 1) consisted of denaturation at 95 °C for 15 min followed by 1 cycle of 95 °C for 20 s, 63°C for 30 s, and 72 °C for 90 s, 2 cycles of 95 °C for 20 s, 62 °C for 30 s, and 72 °C for 90 s, 2 cycles of 95 °C for 20 s, 61 °C for 30 s, and 72 °C for 90 s, followed with 35 cycles of 95 °C for 20 sec, 60 °C for 30 s, and 72 °C for 80 s, and the final extension at 72 °C for 10 min. The nested amplifications used the EHR16SD and pH1492 primers and 1 µL of the primary PCR products as templates. The cycling profile consisted of 95 °C for 15 min; 3 cycles of 95 °C for 20 s, 61 °C for 30 s, and 72 °C for 90 s; 37 cycles of 95 °C for 20 s, 60 °C for 30 s, and 72 °C for 80 s, and a final extension at 72 °C for 10 min. The amplicons were then visualised on an ethidium bromide stained 1% agarose gel. Samples that had the expected band size of 1030 bp were purified and sequenced, as above.

### 2.7. Phylogenetic Analysis

All sequences were edited and aligned, using the MAFFT [29] plugin in Geneious Prime version 2019.0.4 software (created by Biomatters, Auckland, New Zealand). They were subsequently queried in the GenBank nr database (http://www.ncbi.nlm.nih.gov/) using the Basic Local Alignment Search Tool (BLAST) [30]. We constructed maximum likelihood phylogenetic relationships of the study’s gene sequences to those in GenBank using PhyML version 3.0. [31], employing the Akaike information criterion for automatic model selection. Tree topologies were estimated using nearest neighbor interchange improvements over 1000 bootstrap replicates. Trees were visualised in FIGTREE 1.4.2 [32].

### 2.8. Data Management and Analysis

A database consisting of questionnaire and molecular data was established in MS Excel (Microsoft^®^ Excel, Redmond, WA, USA). Individual- and herd-level information, including animal sex, age, animal weight, anaemia status, herd size, village name, distance of homestead from the park’s fence, acaricide use (type and frequency), and infection status, were recorded. The distances of homesteads from the park’s fence were measured using a handheld GPS device. Cattle ages were categorised as ≤2 and >2 years age groups and small (<10 head of cattle) or medium (>10 head of cattle) herd sizes of smallholder farmers. Anaemia status were categorized into anaemic (PCV ≤ 24) and normal (PCV > 24). After checking and variable coding, data were transferred to SPSS version 25 (IBM Corp, Armonk, NY, USA) for statistical analysis the individual animal and herd levels.

The prevalence of each TBP was calculated by dividing the number of positive samples by the total number of animals sampled during study period. Herd level prevalences were obtained by dividing the number of infected herds by the total number of herds tested. A herd was declared as positive for a TBP if one or more animals within the herd tested positive by PCR-HRM analysis.

Associations between prevalence and putative risk factors of TBPs were analysed using univariable logistic regression models. Risk factors associated with infection with specific TBPs were identified by using a multivariable logistic regression model and the strength of their association was assessed using adjusted odds ratios (OR). Variables with a *p*-value ≤ 0.25 in univariable analysis were included in a multivariable logistic model. A backward elimination procedure was used for further selection of variables. The variables were tested for interaction effect using cross-product terms and for multiple-collinearity using the collinearity matrix index before building the final model. The model validity and predictive ability were assessed using the Hosmer–Lemeshow test and receiver operating characteristic (ROC) curve. The confidence level was set at 95% with α = 0.05.

The datasets supporting the conclusion of this article are included within the article and Supplementary Materials files. Nucleotide sequences reported in this article are available via GenBank.

## 3. Results

### 3.1. Pathogen Diversity and Prevalence

Blood from 680 cattle (370 females and 310 males), mainly zebu (*Bos indicus*), from 95 herds were screened for presence of TBPs. The average herd size was seven (ranging from 1–21 cattle per herd) and mainly open grazed as their major form of feeding.

**Figure 2:**
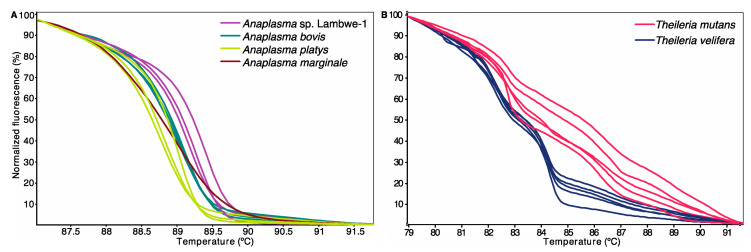
Normalized High-Resolution Melting (HRM) profiles of representative TBP detected (**A**) *Anaplasma* spp. and (**B**) *Theileria* spp. PCR amplicons.

**Figure 3:**
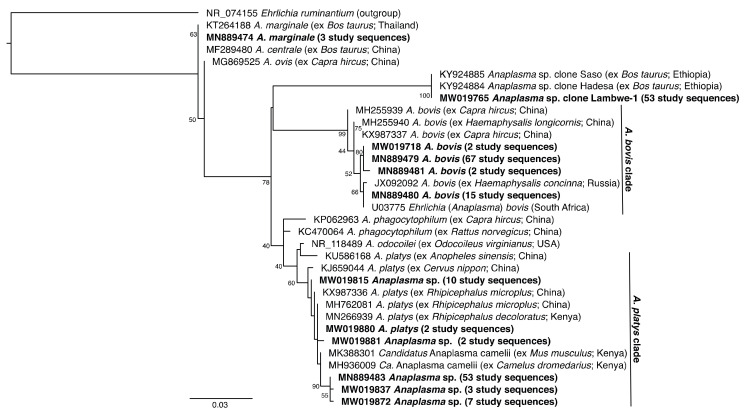
Maximum likelihood phylogeny of 810-bp *Anaplasma* 16S rRNA sequences from cattle blood samples. GenBank accession numbers, species, host species, and country of origin are indicated for each reference 16S rRNA gene sequence. Sequences obtained in this study are highlighted in bold with the number of samples with specific pathogen sequence identified indicated in brackets. The tree is rooted using the *Ehrlichia ruminantium* sequence as an outgroup. Bootstrap values at the major nodes represent percent agreement among 1000 replicates. The branch-length scale represents substitutions per site.

**Figure 4:**
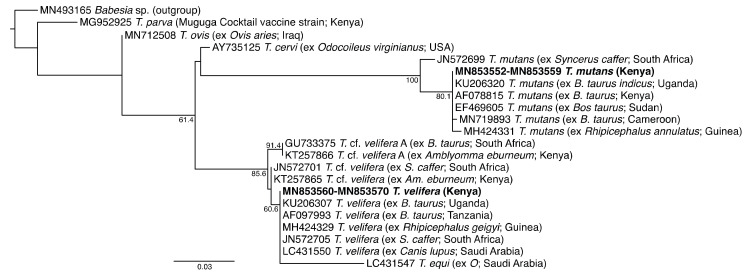
Maximum likelihood phylogeny of 454-bp *Theileria* 18S rRNA sequences from cattle blood samples. GenBank accession numbers, species, host species, and country of origin are indicated for each reference 18S rRNA gene sequence. Sequences obtained in this study are highlighted in bold and countries of origin are indicated in brackets. The tree is rooted using the *Babesia* sp. sequence as an outgroup. Bootstrap values at the major nodes represent percent agreement among 1000 replicates. The branch-length scale represents substitutions per site.

Sequencing of PCR amplicons with expected lengths (16S rRNA gene: ~1000 bp; 18S rRNA gene: 450–500 bp) identified *Anaplasma* and *Theileria* infections with the corresponding melting profiles (Figure 2). In the molecular analysis based on PCR-HRM, 311 (45.7%) and 565 (82%) animals were positive for *Anaplsma* and *Theileria* species, respectively. A total of 303 sample (97.4%, n = 311) and 30 samples (7%, n = 432) of the PCR-HRM positive samples were sequenced for *Anaplasma* and *Theileria* species, respectively. All of these were confirmed as *Anaplasma* and *Theileria* species. Sequence and phylogenetic analysis confirmed the presence of four *Anaplasma* spp. (Figure 3) and two *Theileria* spp. (Figure 4).

We obtained *Anaplasma* sequences that shared 100% identity with *Anaplasma marginale* (deposited GenBank accessions MW019680–MW019681, MN889474; reference GenBank accession MN889474) and > 99% identity with *A. bovis* (deposited GenBank accessions MW019682–MW019761, MN889475–MN889481; reference GenBank accession U03775). We also obtained diverse *A. platys*-like sequences, of which two (deposited GenBank accessions MW019880, MN889489) shared 100% identity with *A. platys* from China (reference GenBank accession MH762081), and 75 (deposited GenBank accessions MW019814–MW019879, MW019881–MW019882, MN889483–MN889488, MN889490) shared > 99% identity with both the *A. platys* reference sequence and *Candidatus* Anaplasma camelii from Kenya (reference GenBank accession MH936009) (Figure 3). These *A. platys*-like sequences are henceforth collectively referred to as *A. platys* clade sequences. We also obtained an *Anaplasma* sp. sequence (Lambwe-1) (deposited GenBank accessions MW019762–MW019813, MN889475) that shared 100% identity to *Anaplasma* sp. clones “Saso” (GenBank accession KY924885) and “Hadessa” (GenBank accession KY924884) from Ethiopia. 

We also identified *Theileria mutans* nucleotide sequences (deposited GenBank accessions MN853552–MN853559) sharing 100% identity with a *T. mutans* from Uganda (GenBank accession KU206320), and *Theileria velifera* sequences (deposited GenBank accessions MN853560–MN853570) sharing 100% identity with *T. velifera* from Tanzania (GenBank accession AF097993) and Uganda (GenBank accession KU206307). All cattle samples tested negative for *Ehrlichia* 16S rRNA, *Rickettsia* 16S rRNA, and *Babesia* 18S rRNA sequences. Additionally, non-specific amplifications using the long *Anaplasma* primers led to sequencing of a *Wolbachia* sp. (deposited GenBank accession MW019883) in four cattle samples.

The apparent prevalence of TBPs at animal- and herd-levels were 78.5% (95% CI: 75.3, 81.5) and 95.8% (95% CI: 91.8, 99.8), respectively (Table 2). The prevalence of *Anaplasma* species was 45.7% (95% CI: 42, 49.5) at animal level and 78.9% (95% CI: 70.8, 87.2) at herd level. For *Theileria*, we recorded a 63.5% (95% CI: 59.9, 67.2) animal-level prevalence and 88.4% (95% CI: 82.0, 94.9) herd-level prevalence. The prevalences of *A. bovis*, *A. marginale*, *Anaplasma* sp. Lambwe-1, and *A. platys* clade sequenses, as well as the two *Theileria* species, are shown in Table 2.

### 3.2. Risk Factors Associated with Anaplasma and Theileria Infections

Results of the univariable logistic regression are summarised in Tables S2 and S3 and Figure 1. Each of the identified TBPs (n = 6) was used as an outcome in independent logistic regression analyses. Our study considered village, herd size, sex, age and distance from park as the risk factors. Small and medium herds were the herd size found in Lambwe Valley, while the distance considered was ≤1 km from the park and >1 km from the park. During the univariate analysis, the first level of each independent variable was used as a reference category. The results indicate that herd size, animal sex, and distance from the park were not significantly associated with the presence of any of the *Anaplasma* and *Theileria* species. However, there was a significant association between the identified *Anaplasma* and *Theileria* species and geospatially clustered villages in which the odds of infection in Kamato, Odelo, and Ruma Pap were significantly higher (Tables S2 and S3).

With the exception of *A. marginale*, all the *Anaplasma* species were detected in all the study villages. The presence of *A. marginale* was confirmed in only two villages, Kamato and Ruma Pap. The odds of *A. platys* clade, *A. bovis*, and *Anaplasma* sp. Lambwe-1 infection were highest in Kamato, Odelo, and Ruma pap (Table S2). The age of the animal was found to be significantly associated with *Theileria* infection; adults had higher odds of infection (OR = 1.8, *p* = 0.014) compared to calves and weaners. In contrast, the odds of *T. velifera* infection were lower in adult cattle (OR = 0.5, *p* = 0.005) compared to calves and weaners.

### 3.3. Prevalence of Co-Infections

Single infections were most common, with a prevalence of 46.9% (Table 3). A total of 215 cattle (31.6%) were co-infected with at least two pathogens. The majority of the co-infections were double infections. The prevalence of double and triple co-infections of TBPs were 29.4% and 2.2%, respectively. *Anaplasma bovis* and *T. velifera* (6.6%), *A. platys* clade and *T. mutans* (5.3%), and *A. platys* clade and *T. velifera* (5.2%) were the three most common double co-infections, while *A. marginale* and *T. velifera* (0.3%) and *T. velifera* and *T. mutans* (1%) co-infections were least common. Cattle with double infection had a combination of one of the identified *Anaplasma* spp. and either *T. velifera* or *T. mutans*. Triple infections consisted mainly of *Anaplasma* sp. Lambwe-1, *A. platys* clade, and *T. mutans* (0.4%); *A. bovis, T. mutans*, and *T. velifera* (0.7%); and *A. platys* clade, *T. mutans*, and *T. velifera* (0.6%).

## 4. Discussion

This study provides insight into the diversity and complex co-occurrence of TBPs in cattle from smallholder farms at the wildlife–livestock interface of Ruma National Park, a protected wildlife area in Kenya. The findings show that cattle are infected with at least four *Anaplasma* species and two *Theileria* species. They further establish that known pathogens such as *A. marginale*, *A. bovis*, *T. mutans*, and *T. velifera* are prevalent in cattle from the study area. Notably, the study provides molecular evidence of *A. platys* and an *Anaplasma* spp. with unknown pathogenicity to livestock or humans.

The overall high prevalence of TBPs found in this study mirrors those recorded in Kenya and other countries in the region within the last five years. We found a TBP prevalence of 78.5% around Ruma. Regionally, reported TBP prevalence in cattle have ranged from 64.5% in Tanzania [33], 75.6% in central Uganda [34] and 89.6% in western Kenya [12], to 96.9% in Ethiopia [35]. The high prevalence of TBPs in cattle around Ruma and the region shows that TBPs could be a significant constraint to animal productivity in the majority of herds.

Three pathogens reported here, *A. bovis*, *A. platys*, and *A. marginale*, are known causative agents of bovine anaplasmosis. The clinical manifestation of infections with these pathogens include anaemia, fever, abortion, weight loss, lymphadenopathy, and death [7]. These symptoms are consistent with those described by local farmers from the study area and suggest that these TBPs could have contributed to the outbreak experienced in the area before the study.

This study reports infections of cattle with *A. bovis* and *A. platys*. Of these, *A. bovis*, was most prevalent (17.4%) and present in all sampled villages. However, this is lower than previously reported in calves (39.9%) in western Kenya [12]. Several tick species, including *Rhipicephalus appendiculatus*, *Amblyomma variegatum*, *Hyalomma* sp., *Rhipicephalus sanguineus*, and *Haemaphysalis* spp. transmit *A. bovis* [36]. Indeed, a study in western Baringo, Kenya, found that about 3% of the *Amblyomma* ticks collected from livestock had *A. bovis* [37]. *Anaplasma platys* commonly infects dog platelets and also causes illness in humans [38]. Matei et al. [39] confirmed that dogs roaming rural communities in islands off the Kenyan coast were infected with *A. platys*. This study, however, found *A. platys*-like DNA in 16.9% of cattle in the study area, a prevalence notably higher than reported in cattle in China (4.35%) [40], Tunisia (3.5%) [41], Brazil (4.75%) [42], and Kenya [43]. However, as there was considerable sequence variation among *A. platys* clade sequences, our findings may represent an unknown diversity of *A. platys*-like pathogens circulating in the region as found in Tunisia [41].

This study recorded a very low prevalence of *A. marginale* (0.6%). In a cross-sectional study conducted in two semi-intensively managed dairy farms in Machakos and Ngong districts, Adjou Moumouni et al. [44] reported *A. marginale* prevalence of up to 32.5%. Similarly, higher *A. marginale* prevalences were reported in Uganda (57%) [45] and Ethiopia (14.5%) [35]. Cattle breeds are differently susceptible to bovine anaplasmosis, with crossbreeds known to have relatively higher prevalences than indigenous cattle breeds [35,45]. The animals in this study were all local breeds.

Although Omondi et al. [37] reported diverse *Anaplasma*, *Ehrlichia*, and *Rickettsia* spp. in *Amblyomma* and *Rhipicephalus* ticks sampled from livestock in adjacent areas of Homa Bay County, *A. marginale* was not identified in the ticks. This complements our data to suggest that *A. marginale* is not prevalent in the region. Similarly, no *A. marginale*, *Ehrlichia* spp., nor *Babesia* spp. were identified in ticks sampled from another wildlife–livestock interface, the Maasai Mara National Reserve [11]. However, *A. marginale*, as well as *T. parva*, *R. africae*, and *Babesia bigemina* have recently been reported in ticks sampled at livestock markets in Busia County [46], approximately 175 km north of Homa Bay County, which suggests that there may be significant regional variation in TBP diversity associated with disparate agro-ecological contexts.

In 11.6% of the cattle sampled, we also identified a distinct *Anaplasma* sp. Lambwe-1 16S rRNA gene sequence that is identical to a recently identified *Anaplasma* sp. (clones “Saso” and “Hadesa”) in Ethiopia [35]. The pathogenicity of this novel *Anaplasma* in cattle and other species and its zoonotic potential remains unknown. Since all of the sampled cattle in this study were apparently healthy, the present study did not evaluate the effect of the identified *Anaplasma* sp. However, the presence of *Anaplasma* sp. Lambwe-1 in the blood was significantly correlated with reduced haematocrite PCV.

We found a higher infection rate with *Theileria* spp. than with *Anaplasma* spp., with *T. mutans* at 25.7% and *T. velifera* at 40%. These pathogens, transmitted by several *Amblyomma* tick species, are less pathogenic and commonly found in cattle [5,47]. Our findings are consistent with most from the horn of Africa and southern Africa, which found higher prevalences of *T. mutans* and *T. velifera* in cattle compared to other TBPs [12,48]. Infections with *T. velifera* in cattle are mostly asymptomatic with animals infected at younger ages having lifelong infections [35,48,49]. Herd prevalences of *Anaplasma* spp. (78.9%) and *Theileria* spp. (88.4%) were higher than their respective individual prevalences of 45.7% and 63.5%. Higher herd prevalence may indicate that the pathogens are more broadly distributed than can be assessed by individual prevalence alone. High herd infection prevalence poses a concern on the herd health of cattle in Lambwe Valley.

Our study provides the first detailed analysis of co-infections in bovine species in Lambwe Valley. The high prevalence of co-infection was mainly due to double infection, the highest rates of which were for *A. bovis* and *T. velifera* (33%), and for *Anaplasma* sp. Lambwe-1 and *T. mutans* (31%) co-infections. The overall prevalence of TBP co-infection was, however, lower than previous studies in western Kenya [12]. Njiiri et al. [12] showed that 87.1% of cattle had mixed infections and that *T. velifera*, *Theileria* sp. clone “sable”, and *T. mutans* were more prominent. In Ethiopia, Hailemariam et al. [35] reported a higher rate (57.9%) of relatively more diverse co-infection ranging from double to sextuple pathogen coinfections. Co-infections can augment transmission of pathogens and/or promote the severity of the diseases [50]. In co-infected animals, the clinical symptoms may not be similar to those in hosts with single infections. Woolhouse et al. [51] demonstrated that mild *Theileria* species reduced the severity of East Coast fever infection in Kenya. This study does not investigate the implication of co-infections to the burden of disease in Lambwe Valley. It however confirms that co-infections do occur in the wildlife–livestock interface and could lead to misdiagnosis and mismanagement of livestock diseases. In addition to co-infections, we found *Wolbachia*, an obligate endobacterial symbiont of bovine *Onchocerca* species that serves as a source of energy for filarial nematodes [52]. Its detection in bovine blood suggests an occult infection of filarial nematodes.

Our risk factor analysis showed that livestock from different villages were at different risks of infection with TBPs. No significant risks were associated with the sex of the animals, the sizes of herds they came from, or the distances of respective homesteads from Ruma National Park. Three village clusters north-east of the park had *Anaplasma* species prevalences > 75%, while three in the west and south-western side had a combined prevalence of 21% for the same pathogens. The type of grazing, tick control interventions applied in different villages, and the composition and abundance of vectors for the pathogens could explain disparities in prevalence between villages. Unlike this study, a previous study found higher prevalence of *A. marginale* in smaller herd sizes compared to medium and larger herds in Peninsular Malaysia [53]. However, the Lambwe Valley communities practice open grazing and cattle share communal watering points. We had hypothesised that cattle populations nearer to the park share more space and potential contact with wildlife than cattle populations further away from the park. However, our analysis showed that the odds of TBPs infection in cattle that reside in close proximity to the park (< 1 km) are not different from those that reside further away. The extensive grazing and livestock management system in Lambwe Valley means that cattle move and the approach of using fixed distances for herds may be inappropriate. This study did not sample wildlife to determine their exposure status with regard to the targeted pathogens; we were therefore unable to confirm a role of wildlife on the observed molecular prevalences.

In conclusion, this study provides an update on the epidemiological status of TBPs circulating in cattle of Lambwe Valley, Kenya. It highlights the high prevalence of infections in the wildlife–livestock interface and reports the existence of highly pathogenic *A. marginale* and *A. bovis*, as well as other novel *Anaplasma* spp., *T. mutans*, and *T. velifera*, with considerable co-infection rates. The results presented offer neither proof of inter-species pathogen transmission nor the incrimination of vectors responsible for the transmission of different pathogens. However, the findings add emphasis on the need to proactively investigate the prevalence and composition of TBPs in wildlife–livestock interfaces.

## Figures and Tables

**Table 1:** Primers used in identification of TBPs.

Target Pathogens	Target Gene	Primer Name	Sequence (5′ to 3′)	Amplicon Size (bp)	Reference
*Rickettsia* spp.	16S rRNA	Rick-F	GAACGCTATCGGTATGCTTAACACA	364	[26]
		Rick-R	CATCACTCACTCGGTATTGCTGGA		
*Ehrlichia* spp.	16S rRNA	EhrlichiaJV F	GCAACCCTCATCCTTAGTTACCA	300	[5]
		EhrlichiaJV R	TGTTACGACTTCACCCTAGTCAC		
*Anaplasma* spp.	16S rRNA	*AnaplasmaJV F*	CGGTGGAGCATGTGGTTTAATTC	300	[5]
		*AnaplasmaJV R*	CGRCGTTGCAACCTATTGTAGTC		
Anaplasmataceae	16S rRNA	EHR16SD	GGTACCYACAGAAGAAGTCC		[27]
		pH1522	AAGGAGGTGATCCAGCCGCA	1060	
		pH1492	GGCTACCTTGTTACGACTT	1030	
*Theileria* and *Babesia* spp.	18S rRNA	RLB F	GAGGTAGTGACAAGAAATAACAATA	450	[28]
		RLB R	*TCTTCGATCCCCTAACTTTC*		

**Table 2:** Individual animal- and herd-level apparent prevalences of TBPs in cattle at the wildlife–livestock interface of Lambwe Valley, western Kenya, 2018/2019.

Pathogen	Individual Prevalence			Herd Prevalence		
	n^a^	Prevalence (%)	95% CI	n^b^	Prevalence (%)	95% CI
*Anaplasma* spp.	311	45.7	42.0, 49.5	75	78.9	70.8, 87.2
*A. bovis*	118	17.4	14.5, 20.2	55	57.9	48.0, 67.8
*A. platys* clade	115	16.9	14.1, 19.7	49	51.6	41.5, 61.6
*A. marginale*	4	0.6	0.0, 1.2	4	4.2	0.2, 8.3
*Anaplasma* sp. Lambwe-1	79	11.6	9.2, 14.0	39	41.1	31.2, 50.9
*Theileria* spp.	432	63.5	59.9, 67.2	84	88.4	82.0, 94.9
*T. velifera*	272	40.0	36.3, 43.7	72	75.8	67.2, 84.4
*T. mutans*	175	25.7	22.5, 29.0	62	65.3	55.7, 74.8
Overall TBPs	680	78.5	75.3, 81.5	91	95.8	91.8, 99.8

n^a^ total individual animals tested positive out of 680, n^b^ total number of herds tested positive out of 95 herds.

**Table 3:** Single and multiple infections detected in cattle in the wildlife–livestock interface in Lambwe Valley, western Kenya.

Pathogen Detected	Number Positive (% Prevalence)	95% CI
Single infections	319 (46.9)	43.7, 50.7
*A. marginale*	2 (0.3)	0.0, 0.7
*A. bovis*	36 (5.3)	3.6, 6.9
*A. platys* clade	35 (5.2)	3.5, 6.8
*Anaplasma* sp. Lambwe-1	29 (4.3)	2.8, 5.8
*T. velifera*	145 (21.0)	18.2, 24.4
*T. mutans*	72 (11.0)	8.3, 12.9
Double infections	200 (29.4)	26.0, 32.8
*A. bovis + T. mutans*	31 (4.6)	3.0, 6.1
*A. bovis + T. velifera*	45 (6.6)	4.8, 8.5
*A. marginale + T. velifera*	2 (0.3)	0.0, 0.7
*A. platys* clade *+ T. mutans*	36 (5.3)	3.5, 6.8
*A. platys* clade *+ T. velifera*	35 (5.2)	3.5, 6.8
*Anaplasma* sp. Lambwe-1 *+ T. velifera*	29 (4.3)	2.8, 5.8
*Anaplasma* sp. Lambwe-1 *+ T. mutans*	17 (2.5)	1.3, 3.7
*T. velifera + T. mutans*	6 (1.0)	0.2, 1.6
Triple infections	15 (2.2)	1.1, 3.3
*A. bovis + A. platys* clade *+ T. mutans*	1 (0.2)	0.0, 0.4
*Anaplasma* sp. Lambwe-1 *+ A. platys* clade *+ T. mutans*	3 (0.4)	0.0, 0.9
*Anaplasma* sp. Lambwe-1 *+ A. platys clade + T. velifera*	1 (0.2)	0.0, 0.4
*A. bovis + T. velifera + T. mutans*	5(0.7)	0.0, 1.4
*A. platys* clade *+ T. mutans + T. velifera*	4 (0.6)	0.0, 1.2
Total	534 (78.5)	75.4, 81.6

## Supplementary Materials

The following are available online at https://www.mdpi.com/2076-2607/8/11/1830/s1, Summary result of the univariable logistic analysis of risk factors with dependent genus *Anaplasma* and genus *Theileria* infection (Table S1), Anaplasma species (Table S2) and *Theileria* species (Table S3) in cattle in the wildlife–livestock interface in Lambwe Valley, western Kenya.

## References

[1] Grootenhuis J.G., Olubayo R.O. (1993). Disease research in the wildlife-livestock interface in Kenya. Vet. Q..

[2] Caron A., Miguel E., Gomo C., Makaya P., Pfukenyi D.M., Foggin C., Hove T., De Garine-Wichatitsky M. (2013). Relationship between burden of infection in ungulate populations and wildlife/livestock interfaces. Epidemiol. Infect..

[3] Jones K.E., Patel N.G., Levy M.A., Storeygard A., Balk D., Gittleman J.L., Daszak P. (2008). Global trends in emerging infectious diseases. Nature.

[4] Miller R.S., Farnsworth M.L., Malmberg J.L. (2013). Diseases at the livestock–wildlife interface: Status, challenges, and opportunities in the United States. Prev. Vet. Med..

[5] Mwamuye M.M., Kariuki E., Omondi D., Kabii J., Odongo D., Masiga D., Villinger J. (2017). Novel *Rickettsia* and emergent tick-borne pathogens: A molecular survey of ticks and tick-borne pathogens in Shimba Hills National Reserve, Kenya. Ticks Tick Borne Dis..

[6] Daszak P., Cunningham A.A., Hyatt A.D. (2000). Emerging infectious diseases of wildlife- threats to biodiversity and human health. Science.

[7] Minjauw B., McLeod A. (2003). Tick-Borne Diseases and Poverty. The Impact of Ticks and Tick-Borne Diseases on the Livelihood of Small-Scale and Marginal Livestock Owners in India and Eastern and Southern Africa.

[8] Ngeranwa J.J., Shompole S.P., Venter E.H., Wambugu A., Crafford J.E., Penzhorn B.L. (2008). Detection of *Anaplasma* antibodies in wildlife and domestic species in wildlife-livestock interface areas of Kenya by major surface protein 5 competitive inhibition enzyme-linked immunosorbent assay. Onderstepoort J. Vet. Res..

[9] Ndeereh D., Muchemi G., Thaiyah A., Otiende M., Angelone-Alasaad S., Jowers M.J. (2017). Molecular survey of *Coxiella burnetii* in wildlife and ticks at wildlife-livestock interfaces in Kenya. Exp. Appl. Acarol..

[10] Omondi D., Masiga D.K., Ajamma Y.U., Fielding B.C., Njoroge L., Villinger J. (2015). Unraveling host-vector-arbovirus interactions by two-gene high resolution melting mosquito bloodmeal analysis in a Kenyan wildlife-livestock interface. PLoS ONE.

[11] Oundo J.W., Villinger J., Jeneby M., Ong’amo G., Otiende M.Y., Makhulu E.E., Musa A.A., Ouso D.O., Wambua L. (2020). Pathogens, endosymbionts, and blood-meal sources of host-seeking ticks in the fast-changing Maasai Mara wildlife ecosystem. PLoS ONE.

[12] Njiiri N.E., Bronsvoort B.M.d., Collins N.E., Steyn H.C., Troskie M., Vorster I., Thumbi S.M., Sibeko K.P., Jennings A., van Wyk I.C. (2015). The epidemiology of tick-borne haemoparasites as determined by the reverse line blot hybridization assay in an intensively studied cohort of calves in western Kenya. Vet. Parasitol..

[13] Kock R.A., Osofsky S.A., Cleaveland S., Karesh W.B., Kock M.D., Nyhus P.J., Star L., Yang A. (2005). What is this infamous “wildlife/livestock disease interface?” A review of current knowledge for the African continent. Conservation and Development Interventions at the Wildlife/Livestock Interface: Implications for Wildlife, Livestock and Human Health.

[14] Morrison W.I., Hemmink J.D., Toye P.G. (2020). *Theileria parva*: A parasite of African buffalo, which has adapted to infect and undergo transmission in cattle. Int. J. Parasitol..

[15] Lwande O.W., Lutomiah J., Obanda V., Gakuya F., Mutisya J., Mulwa F., Michuki G., Chepkorir E., Fischer A., Venter M. (2013). Isolation of tick and mosquito-borne arboviruses from ticks sampled from livestock and wild animal hosts in Ijara District, Kenya. Vector-Borne Zoonotic Dis..

[16] Kenya Wildlife Conservancies Association (2016). State of Wildlife Conservancies in Kenya Report. https://kwcakenya.com/download/state-of-wildlife-conservancies-in-kenya-report/.

[17] Muriuki G.W., Njoka T.J., Reid R.S., Nyariki D.M. (2005). Tsetse control and land-use change in Lambwe Valley, south-western Kenya. Agric. Ecosyst. Environ..

[18] Wellde B.T., Chumo D.A., Reardon M.J., Waema D., Smith D.H., Gibson W.C., Wanyama L., Siongok T.A. (1989). Epidemiology of Rhodesian sleeping sickness in the Lambwe Valley, Kenya. Ann. Trop. Med. Parasitol..

[19] Ogutu J.O., Piepho H.P., Said M.Y., Ojwang G.O., Njino L.W., Kifugo S.C., Wargute P.W. (2016). Extreme wildlife declines and concurrent increase in livestock numbers in Kenya: What are the causes?. PLoS ONE.

[20] Morse S.S., Price-Smith A.T. (2001). Factors in the Emergence of Infectious Diseases.

[21] Otieno D.O., K’Otuto G.O., Jákli B., Schröttle P., Maina J.N., Jung E., Onyango J.C. (2011). Spatial heterogeneity in ecosystem structure and productivity in a moist Kenyan savanna. Plant. Ecol..

[22] Bennett S., Woods T., Liyanage W.M., Smith D.L. (1991). A simplified general method for cluster-sample surveys of health in developing countries. World Health Stat. Q..

[23] Dohoo I.R., Martin S.W., Stryhn H. (2009). Veterinary Epidemiologic Research.

[24] Thrusfield M.V., Christley R. (2018). Veterinary Epidemiology.

[25] Otte M.J., Gumm I.D. (1997). Intra-cluster correlation coefficients of 20 infections calculated from the results of cluster-sample surveys. Prev. Vet. Med..

[26] Nijhof A.M., Bodaan C., Postigo M., Nieuwenhuijs H., Opsteegh M., Franssen L., Jebbink F., Jongejan F. (2007). Ticks and associated pathogens collected from domestic animals in the Netherlands. Vector Borne Zoonotic Dis..

[27] Bastos A.D.S., Mohammed O.B., Bennett N.C., Petevinos C., Alagaili A.N. (2015). Molecular detection of novel Anaplasmataceae closely related to *Anaplasma platys* and *Ehrlichia canis* in the dromedary camel (*Camelus dromedarius*). Vet. Microbiol..

[28] Georges K., Loria G.R., Riili S., Greco A., Caracappa S., Jongejan F., Sparagano O. (2001). Detection of haemoparasites in cattle by reverse line blot hybridisation with a note on the distribution of ticks in Sicily. Vet. Parasitol..

[29] Katoh K., Standley D.M. (2013). MAFFT multiple sequence alignment software version 7: Improvements in performance and usability. Mol. Biol. Evol..

[30] Altschul S.F., Gish W., Miller W., Myers E.W., Lipman D.J. (1990). Basic local alignment search tool. J. Mol. Biol..

[31] Guindon S., Dufayard J.-F., Lefort V., Anisimova M., Hordijk W., Gascuel O. (2010). New algorithms and methods to estimate maximum-likelihood phylogenies: Assessing the performance of PhyML 3.0. Syst. Biol..

[32] Rambaut A. (2014). FigTree.

[33] Ringo A.E., Rizk M.A., Moumouni P.F.A., Liu M., Galon E.M., Li Y., Ji S., Tumwebaze M., Byamukama B., Thekisoe O. (2020). Molecular detection and characterization of tick-borne haemoparasites among cattle on Zanzibar Island, Tanzania. Acta Trop..

[34] Tayebwa D.S., Vudriko P., Tuvshintulga B., Guswanto A., Nugraha A.B., Gantuya S., Batiha G.E.-S., Musinguzi S.P., Komugisha M., Bbira J.S. (2018). Molecular epidemiology of *Babesia* species, *Theileria parva*, and *Anaplasma marginale* infecting cattle and the tick control malpractices in Central and Eastern Uganda. Ticks Tick Borne Dis..

[35] Hailemariam Z., Krucken J., Baumann M., Ahmed J.S., Clausen P.H., Nijhof A.M. (2017). Molecular detection of tick-borne pathogens in cattle from Southwestern Ethiopia. PLoS ONE.

[36] Battilani M., De Arcangeli S., Balboni A., Dondi F. (2017). Genetic diversity and molecular epidemiology of *Anaplasma*. Infect. Genet. Evol..

[37] Omondi D., Masiga D.K., Fielding B.C., Kariuki E., Ajamma Y.U., Mwamuye M.M., Ouso D.O., Villinger J. (2017). Molecular detection of tick-borne pathogen diversities in ticks from livestock and reptiles along the shores and adjacent islands of Lake Victoria and Lake Baringo, Kenya. Front. Vet. Sci..

[38] Arraga-Alvarado C.M., Qurollo B.A., Parra O.C., Berrueta M.A., Hegarty B.C., Breitschwerdt E.B. (2014). Case report: Molecular evidence of *Anaplasma platys* infection in two women from venezuela. Am. J. Trop. Med. Hyg..

[39] Matei I.A., D’Amico G., Yao P.K., Ionică A.M., Kanyari P.W.N., Daskalaki A.A., Dumitrache M.O., Sándor A.D., Gherman C.M., Qablan M. (2016). Molecular detection of *Anaplasma platys* infection in free-roaming dogs and ticks from Kenya and Ivory Coast. Parasit Vectors..

[40] Zhou Z., Li K., Sun Y., Shi J., Li H., Chen Y., Yang H., Li X., Wu B., Li X. (2019). Molecular epidemiology and risk factors of *Anaplasma* spp., *Babesia* spp. and *Theileria* spp. infection in cattle in Chongqing, China. PLoS ONE.

[41] Said M.B., Belkahia H., El Mabrouk N., Saidani M., Alberti A., Zobba R., Cherif A., Mahjoub T., Bouattour A., Messadi L. (2017). *Anaplasma platys*-like strains in ruminants from Tunisia. Infect. Genet. Evol..

[42] André M.R., Calchi A.C., Herrera H.M., de Souza Zanatto D.C., Horta B.d.C.L.S., Tasso J.B., de Souza Ramos I.A., de Mello V.V.C., Machado R.Z. (2020). The co-infection with *Ehrlichia minasensis*, *Anaplasma marginale* and *Anaplasma platys* is not associated with anemia in beef cattle in the Brazilian Pantanal. Vet. Parasitol. Reg. Stud. Reports.

[43] Peter S.G., Aboge G.O., Kariuki H.W., Kanduma E.G., Gakuya D.W., Maingi N., Mulei C.M., Mainga A.O. (2020). Molecular prevalence of emerging *Anaplasma* and *Ehrlichia* pathogens in apparently healthy dairy cattle in peri-urban Nairobi, Kenya. BMC Vet. Res..

[44] Adjou Moumouni P.F., Aboge G.O., Terkawi M.A., Masatani T., Cao S., Kamyingkird K., Jirapattharasate C., Zhou M., Wang G., Liu M. (2015). Molecular detection and characterization of *Babesia bovis*, *Babesia bigemina*, *Theileria* species and *Anaplasma marginale* isolated from cattle in Kenya. Parasit. Vectors.

[45] Byaruhanga C., Collins N.E., Knobel D., Chaisi M.E., Vorster I., Steyn H.C., Oosthuizen M.C. (2016). Molecular investigation of tick-borne haemoparasite infections among transhumant zebu cattle in Karamoja Region, Uganda. Vet. Parasitol. Reg. Stud. Reports.

[46] Chiuya T., Masiga D., Falzon L., Bastos A., Fevre E., Villinger J. (2020). Tick-borne pathogens, including Crimean-Congo haemorrhagic fever virus, at livestock markets and slaughterhouses in western Kenya. Transbound. Emerg. Dis..

[47] Simuunza M., Weir W., Courcier E., Tait A., Shiels B. (2011). Epidemiological analysis of tick-borne diseases in Zambia. Vet. Parasitol..

[48] Moll G., Lohding A., Young A.S., Leitch B.L. (1986). Epidemiology of theileriosis in calves in an endemic area of Kenya. Vet. Parasitol..

[49] Ringo A.E., Aboge G.O., Adjou Moumouni P.F., Lee S.H., Jirapattharasate C., Liu M., Gao Y., Guo H., Zheng W., Efstratiou A. (2019). Molecular detection and genetic characterisation of pathogenic *Theileria*, *Anaplasma* and *Ehrlichia* species among apparently healthy sheep in central and western Kenya. Onderstepoort J. Vet. Res..

[50] Telfer S., Lambin X., Birtles R., Beldomenico P., Burthe S., Paterson S., Begon M. (2010). Species interactions in a parasite community drive infection risk in a wildlife population. Science.

[51] Woolhouse M.E.J., Thumbi S.M., Jennings A., Chase-Topping M., Callaby R., Kiara H., Oosthuizen M.C., Mbole-Kariuki M.N., Conradie I., Handel I.G. (2015). Co-infections determine patterns of mortality in a population exposed to parasite infection. Sci. Adv..

[52] Darby A.C., Armstrong S.D., Bah G.S., Kaur G., Hughes M.A., Kay S.M., Koldkjær P., Rainbow L., Radford A.D., Blaxter M.L. (2012). Analysis of gene expression from the *Wolbachia* genome of a filarial nematode supports both metabolic and defensive roles within the symbiosis. Genome Res..

[53] Ola-Fadunsin S.D., Gimba F.I., Abdullah D.A., Sharma R.S.K., Abdullah F.J.F., Sani R.A. (2018). Epidemiology and risk factors associated with *Anaplasma marginale* infection of cattle in 
Peninsular Malaysia. Parasitol. Int..

